# Integrating pathology, chromosomal instability and mutations for risk stratification in early-stage endometrioid endometrial carcinoma

**DOI:** 10.1186/s13578-020-00486-0

**Published:** 2020-10-22

**Authors:** Yuan Li, Jiaqi Li, Ensong Guo, Jia Huang, Guangguang Fang, Shaohua Chen, Bin Yang, Yu Fu, Fuxia Li, Zizhuo Wang, Rourou Xiao, Chen Liu, Yuhan Huang, Xue Wu, Funian Lu, Lixin You, Ling Feng, Ling Xi, Peng Wu, Ding Ma, Chaoyang Sun, Beibei Wang, Gang Chen

**Affiliations:** 1 National Clinical Research Center of Gynecology and Obstetrics, Tongji Hospital, Tongji Medical College, Huazhong University of Science and Technology, Wuhan, China; 2 Cancer Biology Research Center, Tongji Hospital, Tongji Medical College, Huazhong University of Science and Technology, 1095 Jiefang Anv, Wuhan, 430030 Hubei China; 3 Department of Gynecology,Shenzhen Second People’s Hospital, The First Affiliated Hospital of Shenzhen University, Shenzhen Dapeng New District Maternity & Child Health Hospital, Shenzhen, 518038 China; 4 Department of Gynecology and Obstetrics, The People’s Hospital of Macheng City, Macheng, 438300 China

**Keywords:** Endometrial carcinoma, Chromosomal instability, Histopathology, Molecular pathology, POLE, CTNNB1, Prognostic factor, Risk stratification

## Abstract

**Background:**

Risk stratifications for endometrial carcinoma (EC) depend on histopathology and molecular pathology. Histopathological risk stratification lacks reproducibility, neglects heterogeneity and contributes little to surgical procedures. Existing molecular stratification is useless in patients with specific pathological or molecular characteristics and cannot guide postoperative adjuvant radiotherapies. Chromosomal instability (CIN), the numerical and structural alterations of chromosomes resulting from ongoing errors of chromosome segregation, is an intrinsic biological mechanism for the evolution of different prognostic factors of histopathology and molecular pathology and may be applicable to the risk stratification of EC.

**Results:**

By analyzing CIN25 and CIN70, two reliable gene expression signatures for CIN, we found that EC with unfavorable prognostic factors of histopathology or molecular pathology had serious CIN. However, the POLE mutant, as a favorable prognostic factor, had elevated CIN signatures, and the CTNNB1 mutant, as an unfavorable prognostic factor, had decreased CIN signatures. Only if these two mutations were excluded were CIN signatures strongly prognostic for outcomes in different adjuvant radiotherapy subgroups. Integrating pathology, CIN signatures and POLE/CTNNB1 mutation stratified stageIendometrioid EC into four groups with improved risk prognostication and treatment recommendations.

**Conclusions:**

We revealed the possibility of integrating histopathology and molecular pathology by CIN for risk stratification in early-stage EC. Our integrated risk model deserves further improvement and validation.

## Background

Endometrial carcinoma (EC) is the sixth most common malignant tumor in females worldwide and the second most common in the female reproductive system [1]. The risk stratification of EC is the prerequisite for the accurate evaluation of prognosis, and its ultimate goal is to improve the outcome of patients through the optimization of treatment guidelines. There are currently two kinds of stratification systems, conventional pathology assignment in the guidelines and emerging molecular classification proposed by The Cancer Genome Atlas (TCGA) [2, 3].

In the former system, prognostic factors of histopathology, such as histopathological type, grade, stage, myometrial invasion (MI) and lymphovascular space invasion (LVSI), constitute indications for risk assessment and adjuvant radiotherapy [2]. Numerous retro- and prospective clinical studies have demonstrated that the number and severity of prognostic factors of histopathology positively correlate with the risk of recurrence and the extent of adjuvant therapy in EC [2]. Nevertheless, the lack of consensus among pathologists on the histopathological type and tumor grade assignment has resulted in the same woman receiving different classifications, treatments, and clinical outcomes [4]. In addition to this poor reproducibility of prognostic factors, tremendous diversity in clinical outcomes of patients with the same clinicopathological features suggests that the heterogeneity of EC is ignored in this traditional system [5]. Since most of the prognostic factors of histopathology used for risk stratification are only available after surgery, such as MI and LVSI, this risk model contributes little to decisions regarding surgical procedures.

Existing molecular prognostic factors, such as POLE mutation, copy number variation (CNV) and abnormal expression of mismatch repair proteins, classify EC into four molecular subtypes: POLE-mutant, microsatellite instability (MSI), low copy number variation (CNV-L) and high copy number variation (CNV-H) [3]. In addition, CTNNB1 mutation and L1CAM expression are two independent unfavorable prognostic factors [5–7]. The accurate and objective detection of all these molecular features makes up for the defects of histopathology mentioned above and improves the risk assessment of EC [5, 7]. However, this prognostic refinement, which only exists in patients categorized as “high-intermediate-risk” by the guidelines [5], is not conclusive in “high-risk” EC and is utterly ineffective in “low-risk” disease [8, 9]. In addition to being very expensive and complicated, multiplatform and multimolecular detections also generate some “multiple classifiers” that cannot be stratified accurately and reasonably due to the multiple molecular features in the same patient [9, 10]. More importantly, adjuvant radiotherapy recommendations for patients with specific molecular abnormalities still come from guidelines based on histopathology, and no targeted indication can be used as a Ref. [2]. Therefore, both histopathological and existing molecular stratifications have advantages and disadvantages. We envisioned whether there were more suitable biomarkers and strategies to integrate histopathology and molecular pathology in clinical practice.

Chromosomal instability (CIN), which originates from ongoing errors of chromosome segregation and eventually manifests as both numerical and structural aberrations of chromosomes, including aneuploidy, polyploidy, and CNV [11, 12], exists in approximately 60%–80% of tumors [13]. On the one hand, CIN contributes to adverse phenotypes of tumors, including malignant transformation, poor differentiation, invasion, metastasis, immune evasion and treatment resistance [14–18]. On the other hand, it is the end result of a number of molecular processes, such as mutations in DNA checkpoint genes, microtubule spindle defects, telomere dysfunction and even MSI [19–21]. As a common hallmark and mechanism underlying different phenotypes and molecular features of tumors, CIN may be a common entry point to explore different prognostic factors of histopathology and molecular pathology in EC. Although the respective roles of chromosomal content and chromatin structure in EC have been associated with histopathology and molecular pathology [22–25], the overall impact of the numerical and structural aberrations of chromosomes, which is the significance of CIN, is unclear. Since there is no CIN-specific biomarker for EC, we selected the CIN25 and CIN70 signatures from a pan-cancer genomic instability study to measure the CIN status [26]. Based on the top 25 and 70 genes that have correlations with “total functional aneuploidy” in solid tumors, CIN25 and CIN70 signatures have been proven to fully reflect the numerical and structural complexities of chromosomes and have been successfully used in a broad variety of cancer types and research fields [14, 15, 26–28]. In the present study, our aims were, first, to investigate the interrelationships between the CIN signature and prognostic factors of histopathology or molecular pathology in EC; and second, relying on the integration of the CIN signature and existing stratification systems, to design a novel risk stratification model for improved prognostic refinement and better management of EC.

## Results

### Relationships between CIN and prognostic factors of histopathology in EC

To investigate the CIN reflected by CIN signatures in EC, we first confirmed the difference in CIN signatures between benign and malignant endometria. In the TCGA Uterine Corpus Endometrial Carcinoma (UCEC) cohort, 23 cancer samples had notably increased CIN25 and CIN70 expression levels compared to matched adjacent normal tissues (CIN25: p < 0.001; CIN70: p < 0.001; Additional file 1: Figure S1a). Analysis in the GSE63678 dataset, which contained endometrioid EC (EEC) and four rare pathological types (mixed carcinoma with villoglandular, squamous differentiation, clear cell or papillary serous) gave similar results (CIN25: p = 0.003; CIN70: p = 0.003; Additional file 1: Figure S1b). Additionally, in the GSE17025 dataset, ECs had significantly increased CIN25 and CIN70 compared with benign lesions of the endometrium, including polyps and atrophic, inactive or cystic endometria (CIN25: p < 0.001; CIN70: p < 0.001; Additional file 1: Figure S1c).

The nearly identical outcomes of these detections indicated that abnormal chromosomal stability represented by elevated CIN signatures was a dominant feature of EC. For further exploration of CIN in EC, we then compared CIN signatures among prognostic factors of histopathology.

**Fig. 1:**
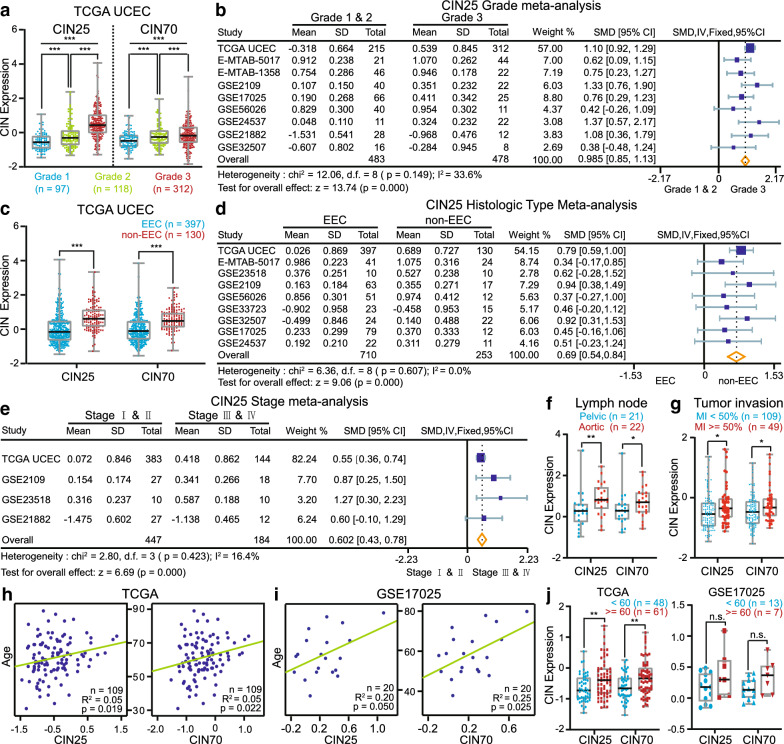
Comparison of CIN signatures among prognostic factors of histopathology. **a** Boxplot of CIN25 and CIN70 expression in Grade 1, Grade 2 and Grade 3 patients from TCGA. If the Levene test for homogeneity demonstrates unequal variances among these three groups, p values are calculated by Welch-corrected ANOVA with Games-Howell post hoc tests. **b** Forest plot comparing CIN25 expression in Grade 1 & 2 versus Grade 3 patients. **c** Boxplot of CIN25 and CIN70 expression in EEC and non-EEC patients from TCGA. **d** Forest plot comparing CIN25 expression in EEC versus non-EEC samples. **e** Forest plot comparing CIN25 expression in Stage I& II versus Stage III & IV patients. In (**b**), (**d**) and (**e**), an inverse variance (IV) fixed effects method is used to meta-analyze the data; squares (blue) represent standardized mean difference (SMD); square size is proportional to weights used in the analysis; bars (gray) represent 95% confidence intervals (CI); diamonds (yellow) represent overall SMD with associated 95% CI (lateral tips). **f** Boxplot of CIN25 and CIN70 expression in Stage IIIC samples with positive pelvic lymph nodes and positive aortic lymph nodes in the TCGA dataset. **g** Boxplot of CIN25 and CIN70 expression in Stage I and Grades 1 & 2 EEC samples with MI < 50% and MI > 50% from the TCGA dataset. **h** Pearson correlation between age and CIN25 or CIN70 expression in Stage I EEC patients with Grades 1 & 2 and MI < 50% from the TCGA dataset. **i** Same as (**h**) but utilizing samples in the GSE17025 dataset. **j** Boxplot of CIN25 and CIN70 expression in patients from (**g**) and (**h**) with age < 60 and > 60. P values presented in (**c**), (**f**), (**g**) and (**j**) are Mann–Whitney test calculations. P values: *p < 0.05, **p < 0.01, ***p < 0.001, n.s. not significantly different

**Fig. 2:**
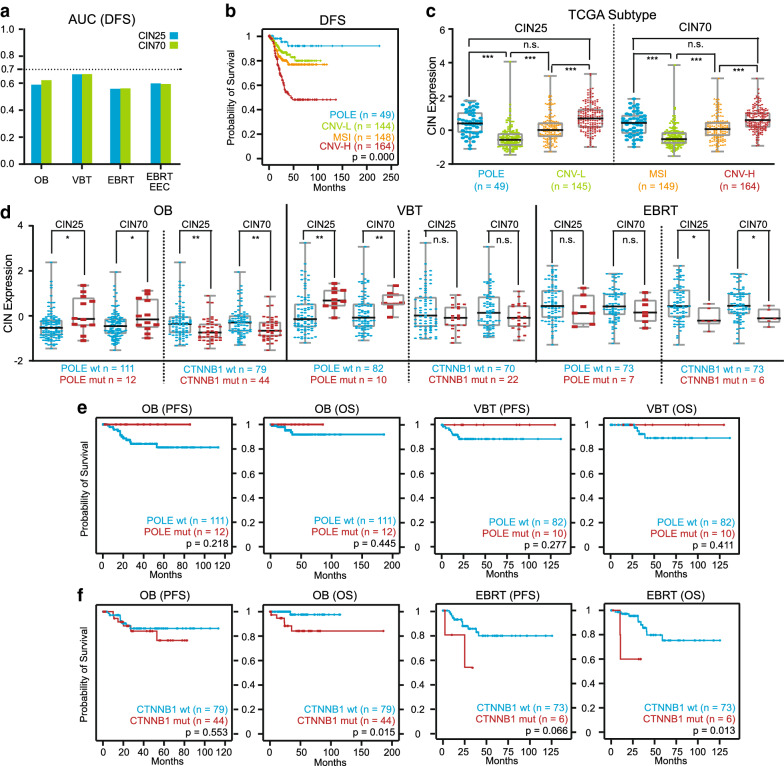
Relationships between CIN and molecular prognostic factors. **a** AUCs for 5-year DFS based on CIN25 and CIN70 in the OB, VBT, EBRT and EBRT EEC subgroups. **b** Kaplan–Meier plot for 5-year DFS based on TCGA molecular subtypes. **c** Boxplot of CIN25 and CIN70 expression in patients belonging to the four TCGA molecular subtypes. The Levene test for homogeneity demonstrated unequal variances among these four groups. P values are calculations of Welch-corrected ANOVA with Games-Howell post hoc tests. **d** Boxplot of CIN25 and CIN70 expression in POLE or CTNNB1 wild-type versus mutant patients from the OB, VBT and EBRT subgroups. P values are Mann–Whitney test calculations. **e** Kaplan–Meier plots for 5-year DFS and 10-year OS based on POLE mutation status in the OB and VBT subgroups. **f** Kaplan–Meier plots for 5-year DFS and 10-year OS based on CTNNB1 mutation status in the OB and EBRT subgroups. P values in (**b**), (**e**) and (**f**) are calculations of the log-rank test. P values: *p < 0.05, **p < 0.01, ***p < 0.001, n.s. not significantly different

First, in the TCGA UCEC cohort, the highest, intermediate and lowest CIN25 and CIN70 values were found in Grade 3, Grade 2 and Grade 1 patients, respectively (CIN25: p < 0.001; CIN70: p < 0.001; Fig. 1a). Meta-analysis including 483 Grade 1 & 2 and 478 Grade 3 patients from 9 EC datasets confirmed the aggravated CIN in Grade 3 (CIN25 SMD: 0.985, 95% confidence interval (CI): 0.85 to 1.13, p = 0.000; CIN70 SMD: 1.009, 95% CI: 0.87 to 1.15, p = 0.000; Fig. 1b and Additional file 1: Figure S1d). This finding suggested that the more serious the CIN, the poorer the tumor differentiation. Second, we observed low-level expression of CIN signatures in EECs and high-level expression in non-EECs from TCGA (CIN25: p < 0.001; CIN70: p < 0.001; Fig. 1c). That unfavorable histopathological type of EC tended to have severe CIN was further demonstrated by meta-analysis comprising 710 EEC and 253 non-EEC patients from 9 datasets (CIN25 SMD: 0.69, 95% CI 0.54 to 0.84, p = 0.000; CIN70 SMD: 0.63, 95% CI 0.48 to 0.78, p = 0.000; Fig. 1d and Additional file 1: Figure S1e). Third, a meta-analysis of 447 Stage I & II patients versus 184 Stage III & IV patients from 4 EC datasets showed that Stage III & IV patients had obviously increased CIN signatures (CIN25 SMD: 0.602, 95% CI 0.43 to 0.78, p = 0.000; CIN70 SMD: 0.592, 95% CI 0.42 to 0.77, p = 0.000; Fig. 1e and Additional file 1: Figure S1f). Furthermore, patients with longer distances of lymph node metastasis (aortic) or deeper MI (MI > 50%) in TCGA had much higher CIN25 and CIN70 (CIN25: p < 0.05; CIN70: p < 0.05; Fig. 1f, g). Thus, the variation in CIN was also an important characteristic of EC progression. Finally, we detected significantly positive correlations between diagnosis age and CIN signatures in EEC patients with Stage I, Grades 1 & 2 and MI < 50% from TCGA (CIN25: R^2^ = 0.05, p = 0.010; CIN70: R^2^ = 0.05, p = 0.011; Fig. 1h). Analysis of the GSE17025 dataset yielded similar results (CIN25: R^2^ = 0.20, p = 0.050; CIN70: R^2^ = 0.25, p = 0.025; Fig. 1i). Patients older than 60 tended to have elevated CIN25 and CIN70 compared with younger patients in TCGA (CIN25: p = 0.0050; CIN70: p = 0.0054; Fig. 2j left). This trend between the two age subgroups did not reach a level of statistical significance in GSE17025, possibly due to insufficient samples (Fig. 2j left).

### Relationships between CIN and prognostic factors of molecular pathology in EC

As all unfavorable prognostic factors of histopathology are tightly associated with aggravated CIN, we speculated whether CIN signatures could be used to conduct risk assessments for different patients in the same adjuvant radiotherapy subgroup classified by the guidelines (observation (OB) subgroup, vaginal brachytherapy (VBT) subgroup and external beam radiation therapy (EBRT) subgroup; "Materials and methods" section and Table 1), thus providing some opportunities to further optimize indications for postoperative adjuvant therapy. Although patients with a high risk of recurrence or progression tended to have high CIN signatures, the areas under the curve (AUCs) for 5-year disease-free survival (DFS) of the OB, VBT, EBRT and EBRT EEC subgroups were not more than 0.67 (Fig. 2a), which was unsatisfactory and prompted us to investigate possible factors for weakening the predictive power of CIN signatures.

**Table 1:** Clinicopathologic parameters according to adjuvant radiotherapy classification in Stage I patients of TCGA UCEC cohort

Prognostic factors	Total	OB	VBT	EBRT	*P*	
	*n *= 294 (100%)	*n*= 123 (42%)	*n* = 92 (31%)	n= 79 (27%)		
Age^a^						
Mean (range)	64 (31–90)	60 (31–89)	66 (35–90)	68 (35–87)	0.000	ANOVA^b^
< 60	97 (33%)	65 (53%)	20 (22%)	12 (15%)	0.000	Pearson Chi^2^
≥ 60	195 (67%)	58 (47%)	71 (78%)	66 (85%)		
Histologic type					0.000	Pearson Chi^2^
Type I, EEC	250 (85%)	123 (100%)	92 (100%)	35 (44%)		
Type II, non-EEC	44 (15%)	0	0	44 (56%)		
Grade					0.000	Pearson Chi^2^
1	76 (26%)	66 (54%)	10 (11%)	0		
2	76 (26%)	57 (46%)	20 (22%)	1(1%)		
3	140 (48%)	0	62 (67%)	78 (99%)		
Stage^c^					0.000	Pearson Chi^2^
IA, MI < 50%	199 (68%)	105 (87%)	62 (67%)	32 (41%)		
IB, MI > 50%	92 (32%)	16 (13%)	30 (33%)	46 (59%)		
CIN expression						
CIN25 Mean (range)	−0.03 (−1.45–3.24)	−0.41 (−1.45–2.38)	0.18 (−1.20–3.24)	0.54 (−1.29–2.23)	0.000	ANOVA
CIN70 Mean (range)	−0.03 (−1.53–3.07)	−0.38 (−1.53–1.95)	0.17 (−1.09–3.07)	0.50 (−1.14–1.86)	0.000	ANOVA
Aneuploidy score	4.82 (0–31)	2.35 (0–20)	4.38 (0–27)	9.18 (0–31)	0.000	ANOVA
FGA	0.15 (0–0.95)	0.08 (0– 0.95)	0.15 (0–0.81)	0.25 (0–0.69)	0.000	ANOVA
Guidelines risk group^d^					0.000	Pearson Chi^2^
Low	105 (36%)	105 (87%)	0	0		
Intermediate	46 (16%)	16 (13%)	30 (33%)	0		
High-intermediate	62 (21%)	0	62 (67%)	0		
High	79 (27%)	0	0	79 (100%)		
TCGA subtype					0.000	Pearson Chi^2^
POLE-mutant	29 (10%)	12 (10%)	10 (11%)	7 (9%)		
MSI	99 (34%)	39 (32%)	37 (40%)	23 (29%)		
CNV Low	101 (34%)	67 (54%)	25 (27%)	9 (11%)		
CNV High	65 (22%)	5 (4%)	20 (22%)	40 (51%)		
Mutation						
PTEN	223 (76%)	111 (90%)	73 (79%)	39 (49%)	0.000	Pearson Chi^2^
FGFR2	59 (20%)	23 (19%)	22 (24%)	14 (18%)	n.s.	Pearson Chi^2^
CTNNB1	72 (24%)	44 (36%)	22 (24%)	6 (8%)	0.000	Pearson Chi^2^
PIK3CA	147 (50%)	67 (54%)	47 (51%)	33 (42%)	n.s.	Pearson Chi^2^
PPP2R1A	40 (14%)	5 (4%)	12 (13%)	23 (29%)	0.000	Pearson Chi^2^

^a^For the two cases without age, one was in VBT group, another was in EBRT group

^b^One-way analysis of variance

^c^For the three cases without accurate MI, two were in OB group, one was in EBRT group

^d^There were two cases in OB group without complete clinicopathological information for guidelines risk assessment

Prognostic factors of molecular pathology became the focus of our investigation. Among the TCGA molecular subtypes of EC except POLE-mutant, CNV-L, MSI and CNV-H had the lowest, intermediate and highest risks of recurrence, respectively, and correspondingly had the lowest, intermediate and highest CIN25 and CIN70 (CIN25: p < 0.001; CIN70: p < 0.001; Fig. 2b, c) [5, 29, 30], which once again implied that CIN might positively correlate with the risk of recurrence in EC. The only exceptional subtype was POLE-mutant, whose prognosis was the best among the four TCGA molecular subtypes, but its CIN signature expression was comparable to that of CNV-H, which had the worst outcome (CIN25: p > 0.05; CIN70: p > 0.05; Fig. 2b, c) [5, 29, 30]. This phenomenon inspired us to explore whether other mutations with prognostic value also had special CIN signatures and in which adjuvant radiotherapy subgroup these special CIN signatures existed. To this end, we compared CIN signatures in wild-type patients with those in POLE, CTNNB1, PTEN, PIK3CA, FGFR2 and PPP2R1A mutant patients from subgroups of OB, VBT, EBRT and ICGC PanCancer Analysis of Whole Genomes (PCAWG) (Fig. 2d and Additional file 2: Figure S2). POLE mutant patients in the OB and VBT subgroups did not relapse or die (Fig. 2e) but had higher expression of CIN25 and CIN70 compared with wild-type patients (CIN25: p < 0.05; CIN70: p < 0.05; Fig. 2d), which might interfere with the risk assessment of CIN signatures. In the OB and EBRT subgroups, the CTNNB1 mutation was another special mutation that had much lower CIN signatures (CIN25: p < 0.05; CIN70: p < 0.05; Fig. 2d and Additional file 2: Figure S2e) but had a much worse prognosis than the wide type (Fig. 2f) [5, 31]. Multivariable Cox models further demonstrated that CTNNB1 mutation was an unfavorable prognostic factor independent of CIN signatures in the OB and EBRT subgroups (Tables 2 and 3). However, this conclusion did not hold in the VBT subgroup, whose CIN signature expression was exactly similar between the CTNNB1 mutant and the wild type (Fig. 2d and Table 2).

**Table 2:** Multivariable analysis on the prognosis role of CIN signatures and CTNNB1 mutation in OB and VBT subgroups without POLE mutation

OB	Disease-free survival			Overall survival		
	*n*	HR (95% CI)	*P*	*n*	HR (95% CI)	*P*
CIN25						
Low	61	1		33	1	
High	50	2.295 (0.749–7.029)	0.146	78	–	0.97
CTNNB1						
Wild type	67	1		67	1	
Mutation	44	1.400 (0.473–4.138)	0.543	44	12.393 (1.325–99.433)	0.022
CIN70						
Low	24	1		44	1	
High	87	–	0.958	67	–	0.966
CTNNB1						
Wild type	67	1		67	1	
Mutation	44	1.576 (0.545–4.554)	0.401	44	12.289 (1.431–105.564)	0.022

VBT	*n*	HR (95% CI)	*P*	*n*	HR (95% CI)	*P*
CIN25						
Low	62	1		63	1	
High	20	6.183 (1.416–26.991)	0.015	19	2.644 (0.372–18.807)	0.331
CTNNB1						
Wild type	60	1		60	1	
Mutation	22	0.562 (0.065–4.838)	0.6	22	–	0.978
CIN70						
Low	50	1		55	1	
High	32	6.032 (1.215–29.949)	0.028	27	2.311 (0.325–16.422)	0.403
CTNNB1						
Wild type	60	1		60	1	
Mutation	22	0.307 (0.038–2.500)	0.27	22	–	0.976

**Table 3:** Multivariable analysis on the prognosis role of CTNNB1 and POLE mutations and CIN signatures in EBRT subgroup

EBRT	Disease-free survival			Overall survival		
	*n*	HR (95% CI)	*P*	*n*	HR (95% CI)	*P*
CIN25						
Low	30	1		11	1	
High	49	2.772 (0.735–10.459)	0.132	68	–	0.977
CTNNB1						
Wild type	73	1		73	1	
Mutation	6	4.907 (1.008–23.880)	0.049	6	6.654 (1.280–34.586)	0.024
POLE						
Wild type	72t	1		72	1	
Mutation	7	0.740 (0.093–5.872)	0.776	7	–	0.983
CIN70						
Low	33	1		12	1	
High	46	3.039 (0.812–11.369)	0.092	67	–	0.976
CTNNB1						
Wild type	73	1		73	1	
Mutation	6	4.889 (1.013–23.602)	0.048	6	6.494 (1.249–33.759)	0.026
POLE						
Wild type	72	1		72	1	
Mutation	7	0.727 (0.092-5.755)	0.763	7	–	0.983

### CIN signatures were prognostic in different adjuvant radiotherapy subgroups of EC

**Fig. 3:**
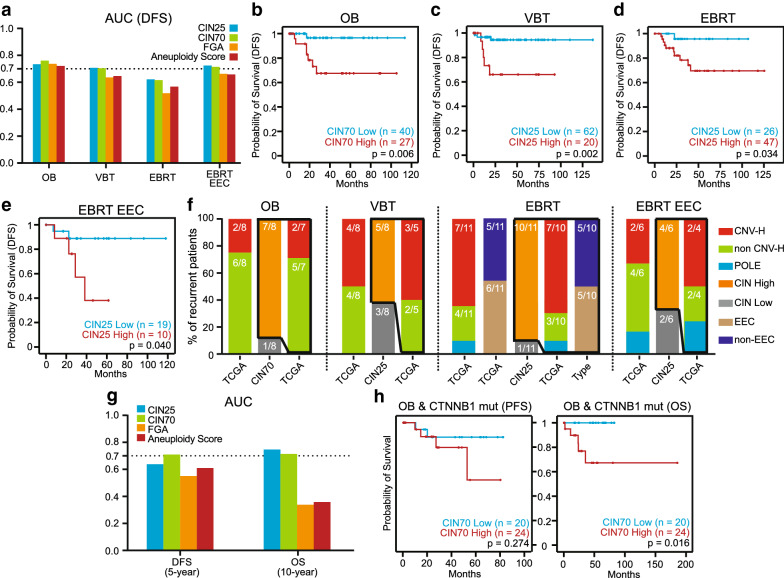
Prognostic significance of CIN signatures in different adjuvant radiotherapy subgroups. **a** AUCs for 5-year DFS based on CIN signatures, FGA and Aneuploidy Score in the OB subgroup excluding mutations of POLE and CTNNB1, in the VBT subgroup excluding POLE-mutant patients and in the EBRT and EBRT EEC subgroups without CTNNB1 mutations. **b** Kaplan–Meier plot for 5-year DFS based on CIN70 in the OB subgroup excluding mutations of POLE and CTNNB1. **c** Kaplan–Meier plot for 5-year DFS based on CIN25 in the VBT subgroup excluding POLE-mutant patients. **d** Kaplan–Meier plot for 5-year DFS based on CIN25 in CTNNB1 wild-type patients from the EBRT subgroup. **e** Kaplan–Meier plot for 5-year DFS based on CIN25 in CTNNB1 wild-type patients from EBRT EEC patients. P values in (**b**–(**e**) are calculations of the log-rank test. **f** Characteristics of recurrent patients in (**b**–(**e**). The cumulative bar chart represents the proportions of TCGA molecular subtypes, the proportions of CIN subgroups and the proportions of histopathological types. **g** AUCs for 5-year DFS and 10-year OS based on CIN signatures, FGA and Aneuploidy Score in CTNNB1-mutant patients from the OB subgroup. **h** Kaplan–Meier plots for 5-year DFS and 10-year overall survival (OS) based on CIN70 in CTNNB1-mutant patients from the OB subgroup. P values are calculations of the log-rank test

Consequently, we tested the prognostic value of CIN signatures in different adjuvant radiotherapy subgroups excluding different special mutations. For the OB subgroup without POLE and CTNNB1 mutations, the AUC based on CIN70 was 0.76 (Fig. 3a), and the CIN70 High group predicted worse DFS than the CIN70 Low group (Fig. 3b). For POLE wild types from the VBT subgroup, the AUC based on CIN25 was 0.71 (Fig. 3a), and the CIN25 High group had a much lower 5-year DFS rate than the CIN25 Low group (Fig. 3c). For CTNNB1 wild types from EBRT and EBRT EEC patients, the AUCs based on CIN25 were 0.62 and 0.72 (Fig. 3a), and the outcomes of the CIN25 High group were much worse than those of the CIN25 Low group (Fig. 3d, e). The predictive powers of the Fraction Genome Altered (FGA) and Aneuploidy Score, two signatures that only evaluate chromosomal content, were far inferior to that of CIN signatures (Fig. 3a). Recurrent patients belonging to different histopathological types or TCGA molecular subtypes can be effectively evaluated by CIN signatures in different adjuvant radiotherapy subgroups (Fig. 3f).

Since the AUCs based on CIN70 for DFS and OS of CTNNB1-mutant patients from the OB subgroup were 0.71 and 0.72 (Fig. 3g), we were curious whether CIN could also play a role in the risk assessment of these patients. Although no statistically significant association between the CIN70 Low group and the CIN70 High group was observed, patients with sufficiently long follow-up in the CIN70 High group exhibited a trend toward worse 5-year DFS (Fig. 3h left). We extended our analysis to 10-year OS and found that the outcome of the CIN70 High group was much worse than that of the CIN70 Low group (Fig. 3h right). We therefore reasoned that the CIN signature could and should be used to stratify the CTNNB1-mutant patients from the OB subgroup.

### Integrated risk assessment for Stage I EEC from TCGA

**Fig. 4:**
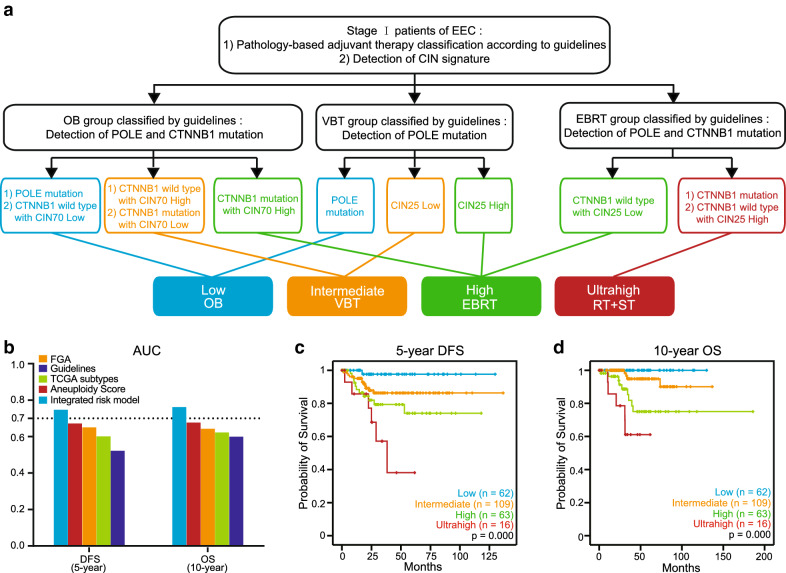
Integrated risk assessment for Stage I EEC. **a** Flow chart of our integrated risk model. **b** AUCs for DFS and OS in Stage I EEC from TCGA based on Guidelines, FGA, Aneuploidy Score, TCGA subtypes and our integrated risk model. **c** Kaplan–Meier plots for 5-year DFS based on integrated risk model in Stage I EEC from TCGA. **d** Kaplan–Meier plots for 10-year OS based on integrated risk model in Stage IEEC from TCGA. P values in (**c**) and (**d**) are calculations of the log-rank test

According to the different effects of CIN signatures, mutations and pathology, a risk assessment model integrating all these factors is proposed in Fig. 4a for Stage I EEC. In this model, four risk profiles (low, intermediate, high and ultrahigh risk) with different prognoses were considered suitable to receive OB, VBT, EBRT and radiotherapy in combination with systemic therapy after surgery. Among the different existing risk stratification systems, our integrated risk model had the highest AUCs for both DFS and OS (AUC for DFS = 0.75, AUC for OS = 0.76; Fig. 4b) and was the only system that had significant prognostic value for both DFS and OS (Fig. 4c, d; Additional file 3: Figure S3).

## Discussion

Through the comparison and meta-analysis of CIN signatures in multiple EC datasets, our study demonstrated that unfavorable prognostic factors of histopathology and molecular pathology, including poor differentiation, non-EEC, advanced disease, deep MI, advanced age, MSI and CNV-H, usually aggravated CIN. A favorable prognostic factor, POLE mutation, and an unfavorable prognostic factor, CTNNB1 mutation, did not follow the above trend. The prognostic value of CIN signatures was well established in different adjuvant radiotherapy subgroups without POLE/CTNNB1 mutations and in CTNNB1 mutant patients from the OB subgroup. An integrated risk model that combines pathology, CIN signatures and mutations was defined for improved prognostic refinement and better management of Stage I EEC.

Most non-EECs are serous and high-grade cancers that exactly have complex aneuploidies and polyploidy [32]; hence, CIN showed consistent changes in fields of histopathological type and tumor differentiation of EC (Fig. 1a–d). At least three potential mechanisms generated by CIN, including the induction of mesenchymal transition, the activation of the STING pathway and immune evasion, may contribute to invasion and metastasis [11], which may explain the high CIN25 and CIN70 in Stage III & IV patients and in patients with deep MI or aortic lymph node metastasis (Fig. 1e–g). Although we cannot verify the CIN status in LVSI-positive patients due to a lack of sufficient pathological information, we speculate that CIN may also increase in LVSI-positive cases since aneuploidy has been correlated with the LVSI of EC [25]. Given the propensity for aging somatic cells to generate unstable chromosomes resulting from gene misexpression, telomeric attrition and senescence failure [33–35], older EC patients were more prone to CIN enrichment (Fig. 1h–j).

Several well-recognized molecular features of EC also have characteristic CIN. One of the final results of CIN is CNV [11]. Therefore, we observed the lowest CIN signature expression in CNV-L and the highest expression in CNV-H (Fig. 2c). The fact that MSI causes some degree of genomic instability and the tendency for MSI to have aggressive phenotypes are two possible reasons for the moderate exacerbation of CIN in MSI patients [18–20, 29, 30]. From CNV-L to MSI and then to CNV-H, as the CIN gradually becomes serious, the risk of recurrence gradually increases (Fig. 2b). In terms of the MSI subtype itself, high CIN signatures were unfavorable prognostic factors [22]. These two pieces of evidence, combined with the fact that CIN signatures did identify recurrent patients who belonged to different TCGA molecular subtypes in each adjuvant radiotherapy subgroup (Fig. 3b–f), implies that CNV-L, MSI, and CNV-H may be pooled together for prognosis evaluation by CIN.

Mutation of POLE causes impaired proofreading activity and DNA repair ability, followed by poor fidelity of DNA replication and severe genomic instability [36, 37]. This makes the CIN of the POLE-mutant subtype roughly the same as that of CNV-H (Fig. 2c). Unlike POLE mutation, however, why the mutation of CTNNB1 is associated with a more stable chromosome status is not clear (Fig. 2d). The aberrant WNT/CTNNB1 pathway in colon cancer always induces CIN [38, 39], so the complete opposite relationship between CTNNB1 mutation and CIN in EC is confusing and interesting. Considering that patients with unstable chromosomes usually have poor clinical outcomes [26], how aggravated CIN produces an excellent prognosis in POLE-mutant patients and how alleviated CIN leads to poor outcomes in CTNNB1-mutant patients is another important issue worthy of further research (Fig. 2d–f; Tables 2 and 3). Serious CIN allows tumors to have different clonal selections in response to various biological stimuli and environmental stresses. However, this selective advantage also has a fitness cost for CIN because the extremely excessive instability of chromosomes is not conducive to the stable survival of the tumor cell itself [11, 14, 27, 40, 41]. For this reason, in addition to the immune activation triggered by POLE-related mutations [42], severe CIN may contribute to the excellent prognosis of POLE-mutant cases. Similarly, CTNNB1-mutant cases, which benefit from the progression and proliferation caused by the activation of WNT/CTNNB1 signaling [6, 43], may protect cells from the adverse effects of this pathway activation with the help of the alleviated CIN. Although this conjecture is still to be confirmed by molecular biology, it may provide CIN-targeted therapeutic strategies for mutation-specific EC.

Based on these data and references, the inherent biological connections between CIN and different prognostic factors of EC suggest that CIN may be a common hallmark in the evolution of different clinicopathological and molecular features, which is the root cause for the success of our integrated risk model (Figs. 3 and 4). From the perspective of risk assessment, the CIN signature, on the one hand, properly addressed the problems of heterogeneity and reproducibility in the conventional pathology system by the precise quantification of CIN status, thereby achieving prognostic refinement. “Multiple classifiers” that cannot be stratified by TCGA subtypes can also obtain accurate and reasonable risk assessments. On the other hand, the prognostic refinement achieved by CIN signatures existed in all adjuvant radiotherapy subgroups in the guidelines, which means that CIN may have more universal applications compared to other risk stratification systems such as TCGA subtypes, FGA and Aneuploidy Score. From a therapeutic point of view, high concordance of molecular alterations between curettage samples and hysterectomy specimens from EC suggested the potential for CIN signatures to guide surgical management [24, 44]. More importantly, because the accurate risk stratification accomplished by the CIN signature presupposed the adjuvant radiotherapy classification based on the guidelines, the treatment recommendations obtained from our integrated risk model may be an intact inheritance of and effective supplement to the indications for postoperative radiotherapy in the guidelines. In summary, the intrinsic relationships between CIN and clinicopathological or molecular features make CIN a bridge that comprehensively integrates histopathology and molecular pathology, which is difficult for other biomarkers to achieve.

To further refine our integrated risk model, we face two outstanding challenges. First, the CIN signature should be optimized on the basis of CIN25 and CIN70. Different adjuvant radiotherapy subgroups in the guidelines have different clinicopathological and molecular features (Table 1), which leads to the same CIN signature having different capabilities of risk assessment in different adjuvant radiotherapy subgroups. For the same adjuvant radiotherapy subgroup, different CIN signatures also have different risk assessment capabilities. Consequently, we believe that it is necessary to improve the respective CIN signatures for the OB, OB & CTNNB1 mutation, VBT and EBRT EEC patients classified by the guidelines to realize the full potential of CIN. Second, the relationships between CIN and LVSI or several EC prognostic factors assessed by immunohistochemistry [45], such as L1CAM, ER and PR, remain to be explored. It is unclear whether these features are still independent prognostic factors in our integrated model. We look forward to high-quality retrospective studies with mature long-term follow-up data and large sample sizes that will meet these two challenges and provide a solid foundation for future clinical applications.

## Conclusions

Overall, except for POLE and CTNNB1 mutations, serious CIN represented by increased CIN25 and CIN70 are characteristic of unfavorable prognostic factors in EC. Integration of pathology, CIN signatures and mutation of POLE/CTNNB1 in Stage I EEC leads to improved prognostic refinement with potential clinical utility. Our integrated risk model holds promise to reduce both overtreatment and undertreatment and deserves further validation and improvement.

## Materials and methods

### Data collection

Clinical information, gene expression (Z-score), mutation profiles, FGA and Aneuploidy Score of the TCGA UCEC cohort are available at cBioPortal for Cancer Genomics [46]. RNAseq data (FPKM-UQ) of EC samples and matched adjacent normal tissues were downloaded from the TCGA data portal [47]. Mutation profiles and RNAseq data (FPKM-UQ) of EEC samples in the ICGC PCAWG project were downloaded from the ICGC data portal [48, 49]. E-MTAB-1358 and E-MTAB-5018 were downloaded from the ArrayExpress database [50–52]. GSE2109, GSE23518, GSE56026, GSE33723, GSE32507, GSE17025, GSE24537, GSE21882 and GSE63678 were downloaded from the Gene Expression Omnibus (GEO) database [53–61]. Each dataset downloaded from ArrayExpress and GEO databases was standardized using the Z-score transformation before calculations of CIN signatures. CIN25 and CIN70 signatures for each sample were the average expressions of the 25 and 70 genes identified by Cart et al. (Additional file 4: Table S1) [26] and were compared among prognostic factors of histopathology or molecular pathology. The clinicopathological information for each dataset is shown in the meta-analysis.

### Adjuvant radiotherapy classification for StageI patients in the TCGA UCEC cohort

There were three adjuvant therapeutic strategies after surgery for stage I EC patients, namely, observation (OB), vaginal brachytherapy (VBT), and external beam radiation therapy (EBRT). Indications for the three adjuvant radiotherapies in the guidelines of ESMO-ESGO-ESTRO were based on six established clinicopathological risk factors, including age, histologic type, grade, stage, MI, and LVSI [2]. LVSI was missing in the TCGA UCEC cohort; therefore, we had to conduct the classification with the other five risk factors. The OB subgroup in the guidelines was defined as a) stage IA EEC with Grades 1 & 2 and b) stage IB EEC with Grades 1 & 2 and less than 60 years old. Patients in the EBRT subgroup followed the following criteria: a) stage IB EEC with Grade 3; b) stage I non-EEC. The VBT subgroup consisted of the remaining patients, including a) stage IB EEC with Grades 1 & 2 and age > 60; b) stage IA EEC with Grade 3. Patients who did not have complete or accurate information for classification and survival analysis, who had other malignancies or who had positive surgical margins were excluded. Ultimately, there were 123, 92 and 79 patients in the OB, VBT and EBRT subgroups, respectively. Detailed information is presented in Table 1.

### Survival analysis for different adjuvant radiotherapy subgroups

In the OB, VBT, EBRT and EBRT EEC subgroups, AUC and optimal cutoff values based on CIN25 and CIN70 signatures, FGA and Aneuploidy Score were determined by the time-dependent receiver operating curve using the “survivalROC” package on the R platform. Kaplan–Meier curves and log-rank tests were carried out to predict 5-year DFS and 10-year OS based on the optimal cutoff values or mutation status of different subgroups. Cox proportional hazards models were used to evaluate the prognostic value of mutations and CIN signatures. Covariates violating the proportional hazards assumption were added as time-dependent covariates in the Cox regression models.

### Statistical analysis

If not specified otherwise, comparisons among clinicopathological features and among molecular alterations were tested using the Chi square test for categorical variables and using the Mann–Whitney test or one-way analysis of variance (ANOVA) in continuous cases. SPSS 21.0 and GraphPad Prism 8 software programs were used to perform statistical and survival analyses and to plot graphs. Meta-analysis was performed using STATA 12.0. Data in this article are presented as the mean ± S.D., and p values were based on two-sided tests with < 0.05 considered statistically significant.

## Supplementary information

**Additional file 1: Figure S1.** Comparison of CIN signatures among prognostic factors of histopathology. **a** Paired scatter plots of CIN25 (left) and CIN70 (right) expression profiles comparing endometrial carcinomas (red) with paired adjacent normal tissues (blue) from TCGA dataset (n = 23). Each pair of dots indicates the amount of CIN25 or CIN70 expression for a particular patient. P values represent paired *t* test calculations. **b** Boxplots of CIN25 (left) and CIN70 (right) expression in normal endometria (blue) and endometrial carcinomas (red). Each dot indicates the amount of CIN25 or CIN70 expression for a particular sample in the GSE63678 dataset. (**c**) Same as (**b**) but comparing benign lesions (blue) with endometrial carcinomas (red) from the GSE17025 dataset. P values presented in (**b**) and (**c**) are Mann–Whitney test calculations. **d** Forest plot comparing CIN70 expression in Grade 1 & 2 versus Grade 3 patients. **e** Forest plot comparing CIN70 expression in EEC versus non-EEC samples. **f** Forest plot comparing CIN70 expression in Stage I & II versus Stage III & IV patients. In (**d**), (**e**) and (**f**), an inverse variance (IV) fixed effects method was used to meta-analyze the data; squares (blue) represent standardized mean difference (SMD); square size is proportional to weights used in the analysis; bars (gray) represent 95% confidence intervals (CI); diamonds (yellow) represent overall SMD with associated 95% CI (lateral tips). P values: *p < 0.05, **p < 0.01, ***p < 0.001.

**Additional file 2: Figure S2.** Relationships between CIN and molecular prognostic factors. **a** Boxplot of CIN25 and CIN70 expression in PTEN wild-type (blue) versus mutant patients (red) from the OB, VBT and EBRT groups. **b** Boxplot of CIN25 and CIN70 expression in PIK3CA wild-type (blue) versus mutant patients (red) from the OB, VBT and EBRT groups. **c** Boxplot of CIN25 and CIN70 expression in FGFR2 wild-type (blue) versus mutant patients (red) from the OB, VBT and EBRT groups. **d** Boxplot of CIN25 and CIN70 expression in PPP2R1A wild-type (blue) versus mutant patients (red) from the OB, VBT and EBRT groups. **e** Boxplot of CIN25 and CIN70 expression in wild-type POLE, CTNNB1, PTEN, PIK3CA, FGFR2 and PPP2R1A (blue) versus mutant patients (red) from the PCAWG EEC cohort. P values in (**a**)–(**e**) are Mann–Whitney test calculations (*p < 0.05, **p < 0.01, ***p < 0.001, n.s. not significantly different).

**Additional file 3: Figure S3.** Kaplan–Meier plot for DFS and OS in Stage I EEC from TCGA based on Guidelines, FGA, Aneuploidy Score and TCGA subtypes. **a** 5-year PFS. **b** 10-year OS. Patients are grouped by quartiles of FGA and Aneuploidy Score. P values for (**a**) and (**b**) are calculations of the log-rank test.

**Additional file 4: Table S1.** Genes in CIN25 and CIN70.

## Data Availability

All data generated or analysed during this study are included in this published article and its supplementary information files.

## Abbreviations

CIN: Chromosomal instability
EC: Endometrial carcinoma
TCGA: The Cancer Genome Atlas
MI: Myometrial invasion
LVSI: Lymphovascular space invasion
CNV: Copy number variation
MSI: Microsatellite instability
CNV-L: Low copy number variation
CNV-H: High copy number variation
UCEC: Uterine Corpus Endometrial Carcinoma
EEC: Endometrioid endometrial carcinoma
OB: Observation
VBT: Vaginal brachytherapy
EBRT: External beam radiation therapy
AUC: area under the curve
DFS: Disease-free survival
OS: Overall survival

## References

[1] Bray F, Ferlay J, Soerjomataram I, Siegel RL, Torre LA, Jemal A (2018). Global cancer statistics 2018: GLOBOCAN estimates of incidence and mortality worldwide for 36 cancers in 185 countries. CA Cancer J Clin.

[2] Colombo N, Creutzberg C, Amant F, Bosse T, Gonzalez-Martin A, Ledermann J, et al. ESMO-ESGO-ESTRO Consensus Conference on Endometrial Cancer: diagnosis, treatment and follow-up. Ann Oncol. 2016;27(1):16-41. 10.1093/annonc/mdv484 26634381

[3] Cancer Genome Atlas Research N, Kandoth C, Schultz N, Cherniack AD, Akbani R, Liu Y, et al. Integrated genomic characterization of endometrial carcinoma. Nature. 2013. 497(7447):67-73. 10.1038/nature12113 PMC3704730 23636398

[4] Clarke BA, Gilks CB (2010). Endometrial carcinoma: controversies in histopathological assessment of grade and tumour cell type. J Clin Pathol.

[5] Stelloo E, Nout RA, Osse EM, Jurgenliemk-Schulz IJ, Jobsen JJ, Lutgens LC (2016). Improved risk assessment by integrating molecular and clinicopathological factors in early-stage endometrial cancer-combined analysis of the PORTEC cohorts. Clin Cancer Res.

[6] Liu Y, Patel L, Mills GB, Lu KH, Sood AK, Ding L, et al. Clinical significance of CTNNB1 mutation and Wnt pathway activation in endometrioid endometrial carcinoma. J Natl Cancer Inst. 2014. 106(9). 10.1093/jnci/dju245 PMC4200060 25214561

[7] Zeimet AG, Reimer D, Huszar M, Winterhoff B, Puistola U, Azim SA (2013). L1CAM in early-stage type I endometrial cancer: results of a large multicenter evaluation. J Natl Cancer Inst.

[8] Stelloo E, Bosse T, Nout RA, MacKay HJ, Church DN, Nijman HW (2015). Refining prognosis and identifying targetable pathways for high-risk endometrial cancer; a TransPORTEC initiative. Mod Pathol.

[9] McAlpine J, Leon-Castillo A, Bosse T (2018). The rise of a novel classification system for endometrial carcinoma; integration of molecular subclasses. J Pathol.

[10] Schultheis AM, Martelotto LG, De Filippo MR, Piscuglio S, Ng CK, Hussein YR (2016). TP53 Mutational Spectrum in Endometrioid and Serous Endometrial Cancers. Int J Gynecol Pathol.

[11] Turajlic S, Sottoriva A, Graham T, Swanton C (2019). Resolving genetic heterogeneity in cancer. Nat Rev Genet.

[12] Sansregret L, Vanhaesebroeck B, Swanton C (2018). Determinants and clinical implications of chromosomal instability in cancer. Nat Rev Clin Oncol.

[13] Carter SL, Cibulskis K, Helman E, McKenna A, Shen H, Zack T (2012). Absolute quantification of somatic DNA alterations in human cancer. Nat Biotechnol.

[14] Endesfelder D, McGranahan N, Birkbak NJ, Szallasi Z, Kschischo M, Graham TA (2011). A breast cancer meta-analysis of two expression measures of chromosomal instability reveals a relationship with younger age at diagnosis and high risk histopathological variables. Oncotarget.

[15] Swanton C, Nicke B, Schuett M, Eklund AC, Ng C, Li Q (2009). Chromosomal instability determines taxane response. Proc Natl Acad Sci USA.

[16] Turajlic S, Swanton C (2016). Metastasis as an evolutionary process. Science.

[17] Bakhoum SF, Cantley LC (2018). The multifaceted role of chromosomal instability in cancer and its microenvironment. Cell.

[18] Rajagopalan H, Nowak MA, Vogelstein B, Lengauer C (2003). The significance of unstable chromosomes in colorectal cancer. Nat Rev Cancer.

[19] Li LS, Kim NG, Kim SH, Park C, Kim H, Kang HJ (2003). Chromosomal imbalances in the colorectal carcinomas with microsatellite instability. Am J Pathol.

[20] Trautmann K, Terdiman JP, French AJ, Roydasgupta R, Sein N, Kakar S (2006). Chromosomal instability in microsatellite-unstable and stable colon cancer. Clin Cancer Res.

[21] Grady WM (2004). Genomic instability and colon cancer. Cancer Metastasis Rev.

[22] Proctor L, Pradhan M, Leung S, Cheng A, Lee CH, Soslow RA (2017). Assessment of DNA Ploidy in the ProMisE molecular subgroups of endometrial cancer. Gynecol Oncol.

[23] Hveem TS, Njolstad TS, Nielsen B, Syvertsen RA, Nesheim JA, Kjaereng ML (2017). Changes in chromatin structure in curettage specimens identifies high-risk patients in endometrial cancer. Cancer Epidemiol Biomarkers Prev.

[24] Njolstad TS, Trovik J, Hveem TS, Kjaereng ML, Kildal W, Pradhan M (2015). DNA ploidy in curettage specimens identifies high-risk patients and lymph node metastasis in endometrial cancer. Br J Cancer.

[25] Song T, Lee JW, Choi CH, Kim TJ, Bae DS, Sung CO (2012). Ploidy and S-phase fraction are correlated with lymphovascular space invasion that is predictive of outcomes in endometrial cancer. Int J Clin Oncol.

[26] Carter SL, Eklund AC, Kohane IS, Harris LN, Szallasi Z (2006). A signature of chromosomal instability inferred from gene expression profiles predicts clinical outcome in multiple human cancers. Nat Genet.

[27] Birkbak NJ, Eklund AC, Li Q, McClelland SE, Endesfelder D, Tan P (2011). Paradoxical relationship between chromosomal instability and survival outcome in cancer. Cancer Res.

[28] Decaux O, Lode L, Magrangeas F, Charbonnel C, Gouraud W, Jezequel P (2008). Prediction of survival in multiple myeloma based on gene expression profiles reveals cell cycle and chromosomal instability signatures in high-risk patients and hyperdiploid signatures in low-risk patients: a study of the Intergroupe Francophone du Myelome. J Clin Oncol.

[29] Talhouk A, McConechy MK, Leung S, Yang W, Lum A, Senz J (2017). Confirmation of ProMisE: A simple, genomics-based clinical classifier for endometrial cancer. Cancer.

[30] Kommoss S, McConechy MK, Kommoss F, Leung S, Bunz A, Magrill J (2018). Final validation of the ProMisE molecular classifier for endometrial carcinoma in a large population-based case series. Ann Oncol.

[31] Wortman BG, Bosse T, Nout RA, Lutgens L, van der Steen-Banasik EM, Westerveld H (2018). Molecular-integrated risk profile to determine adjuvant radiotherapy in endometrial cancer: evaluation of the pilot phase of the PORTEC-4a trial. Gynecol Oncol.

[32] Taylor AM, Shih J, Ha G, Gao GF, Zhang X, Berger AC, et al. Genomic and Functional Approaches to Understanding Cancer Aneuploidy. Cancer Cell. 2018. 33(4):676-89 e3. 10.1016/j.ccell.2018.03.007 PMC6028190 29622463

[33] Geigl JB, Langer S, Barwisch S, Pfleghaar K, Lederer G, Speicher MR (2004). Analysis of gene expression patterns and chromosomal changes associated with aging. Cancer Res.

[34] Hackett JA, Feldser DM, Greider CW (2001). Telomere dysfunction increases mutation rate and genomic instability. Cell.

[35] Artandi SE, Chang S, Lee SL, Alson S, Gottlieb GJ, Chin L (2000). Telomere dysfunction promotes non-reciprocal translocations and epithelial cancers in mice. Nature.

[36] Henninger EE, Pursell ZF (2014). DNA polymerase epsilon and its roles in genome stability. IUBMB Life.

[37] Tubbs A, Nussenzweig A (2017). Endogenous DNA damage as a source of genomic instability in cancer. Cell.

[38] Hadjihannas MV, Bruckner M, Jerchow B, Birchmeier W, Dietmaier W, Behrens J (2006). Aberrant Wnt/beta-catenin signaling can induce chromosomal instability in colon cancer. Proc Natl Acad Sci USA.

[39] Hadjihannas MV, Behrens J (2006). CIN By WNT: growth pathways, mitotic control and chromosomal instability in cancer. Cell Cycle.

[40] Jamal-Hanjani M, A’Hern R, Birkbak NJ, Gorman P, Gronroos E, Ngang S (2015). Extreme chromosomal instability forecasts improved outcome in ER-negative breast cancer: a prospective validation cohort study from the TACT trial. Ann Oncol.

[41] Roylance R, Endesfelder D, Gorman P, Burrell RA, Sander J, Tomlinson I (2011). Relationship of extreme chromosomal instability with long-term survival in a retrospective analysis of primary breast cancer. Cancer Epidemiol Biomarkers Prev.

[42] van Gool IC, Bosse T, Church DN (2016). POLE proofreading mutation, immune response and prognosis in endometrial cancer. Oncoimmunology.

[43] Klaus A, Birchmeier W (2008). Wnt signalling and its impact on development and cancer. Nat Rev Cancer.

[44] Stelloo E, Nout RA, Naves LC, Ter Haar NT, Creutzberg CL, Smit VT (2014). High concordance of molecular tumor alterations between pre-operative curettage and hysterectomy specimens in patients with endometrial carcinoma. Gynecol Oncol.

[45] Karnezis AN, Leung S, Magrill J, McConechy MK, Yang W, Chow C (2017). Evaluation of endometrial carcinoma prognostic immunohistochemistry markers in the context of molecular classification. J Pathol Clin Res.

[46] cBioPortal for Cancer Genomics Available from: https://www.cbioportal.org/.

[47] TCGA data portal Available from: www.portal.gdc.cancer.gov/.

[48] Consortium ITP-CAoWG. Pan-cancer analysis of whole genomes. Nature. 2020. 578(7793):82-93. 10.1038/s41586-020-1969-6 PMC7025898 32025007

[49] ICGC data portal https://dcc.icgc.org/.

[50] Krakstad C, Birkeland E, Seidel D, Kusonmano K, Petersen K, Mjos S (2012). High-throughput mutation profiling of primary and metastatic endometrial cancers identifies KRAS, FGFR2 and PIK3CA to be frequently mutated. PLoS ONE.

[51] Kusonmano K, Halle MK, Wik E, Hoivik EA, Krakstad C, Mauland KK (2018). Identification of highly connected and differentially expressed gene subnetworks in metastasizing endometrial cancer. PLoS ONE.

[52] ArrayExpress. https://www.ebi.ac.uk/arrayexpress/.

[53] Chiyoda T, Tsuda H, Tanaka H, Kataoka F, Nomura H, Nishimura S (2012). Expression profiles of carcinosarcoma of the uterine corpus-are these similar to carcinoma or sarcoma?. Genes Chromosomes Cancer.

[54] Risinger JI, Allard J, Chandran U, Day R, Chandramouli GV, Miller C (2013). Gene expression analysis of early stage endometrial cancers reveals unique transcripts associated with grade and histology but not depth of invasion. Front Oncol.

[55] Mhawech-Fauceglia P, Wang D, Kesterson J, Syriac S, Clark K, Frederick PJ (2011). Gene expression profiles in stage I uterine serous carcinoma in comparison to grade 3 and grade 1 stage I endometrioid adenocarcinoma. PLoS ONE.

[56] Levan K, Partheen K, Osterberg L, Olsson B, Delle U, Eklind S (2010). Identification of a gene expression signature for survival prediction in type I endometrial carcinoma. Gene Expr.

[57] Mhawech-Fauceglia P, Wang D, Kesterson J, Clark K, Monhollen L, Odunsi K (2010). Microarray analysis reveals distinct gene expression profiles among different tumor histology, stage and disease outcomes in endometrial adenocarcinoma. PLoS ONE.

[58] Romero-Perez L, Castilla MA, Lopez-Garcia MA, Diaz-Martin J, Biscuola M, Ramiro-Fuentes S (2013). Molecular events in endometrial carcinosarcomas and the role of high mobility group AT-hook 2 in endometrial carcinogenesis. Hum Pathol.

[59] Pappa KI, Polyzos A, Jacob-Hirsch J, Amariglio N, Vlachos GD, Loutradis D (2015). Profiling of discrete gynecological cancers reveals novel transcriptional modules and common features shared by other cancer types and embryonic stem cells. PLoS ONE.

[60] Kharma B, Baba T, Matsumura N, Kang HS, Hamanishi J, Murakami R (2014). STAT1 drives tumor progression in serous papillary endometrial cancer. Cancer Res.

[61] Gene Expression Omnibus Available from: https://www.ncbi.nlm.nih.gov/gds.

