# Supplementary figures and images for: Integrating pathology, chromosomal instability and mutations for risk stratification in early-stage endometrioid endometrial carcinoma
Source: Cell Biosci. 2020 Oct 22;10:122. doi: 10.1186/s13578-020-00486-0 (PMC7583263; doi:10.1186/s13578-020-00486-0)

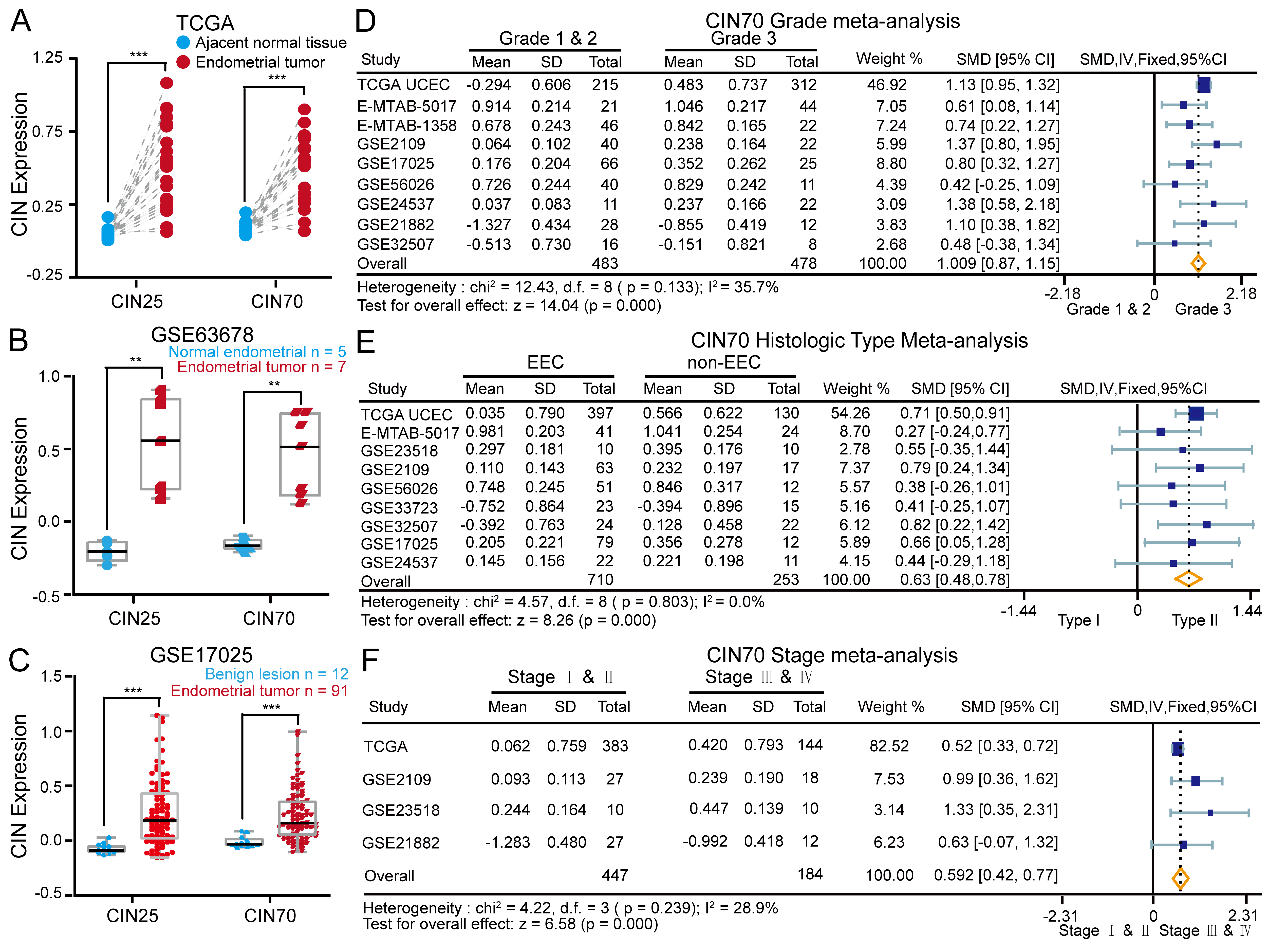

Supplement: Supplementary file 1 — Additional file 1: Figure S1. Comparison of CIN signatures among prognostic factors of histopathology. a Paired scatter plots of CIN25 (left) and CIN70 (right) expression profiles comparing endometrial carcinomas (red) with paired adjacent normal tissues (blue) from TCGA dataset (n = 23). Each pair of dots indicates the amount of CIN25 or CIN70 expression for a particular patient. P values represent paired t test calculations. b Boxplots of CIN25 (left) and CIN70 (right) expression in normal endometria (blue) and endometrial carcinomas (red). Each dot indicates the amount of CIN25 or CIN70 expression for a particular sample in the GSE63678 dataset. (c) Same as (b) but comparing benign lesions (blue) with endometrial carcinomas (red) from the GSE17025 dataset. P values presented in (b) and (c) are Mann–Whitney test calculations. d Forest plot comparing CIN70 expression in Grade 1 & 2 versus Grade 3 patients. e Forest plot comparing CIN70 expression in EEC versus non-EEC samples. f Forest plot comparing CIN70 expression in Stage I & II versus Stage III & IV patients. In (d), (e) and (f), an inverse variance (IV) fixed effects method was used to meta-analyze the data; squares (blue) represent standardized mean difference (SMD); square size is proportional to weights used in the analysis; bars (gray) represent 95% confidence intervals (CI); diamonds (yellow) represent overall SMD with associated 95% CI (lateral tips). P values: *p < 0.05, **p < 0.01, ***p < 0.001. [file 13578_2020_486_MOESM1_ESM.tif]

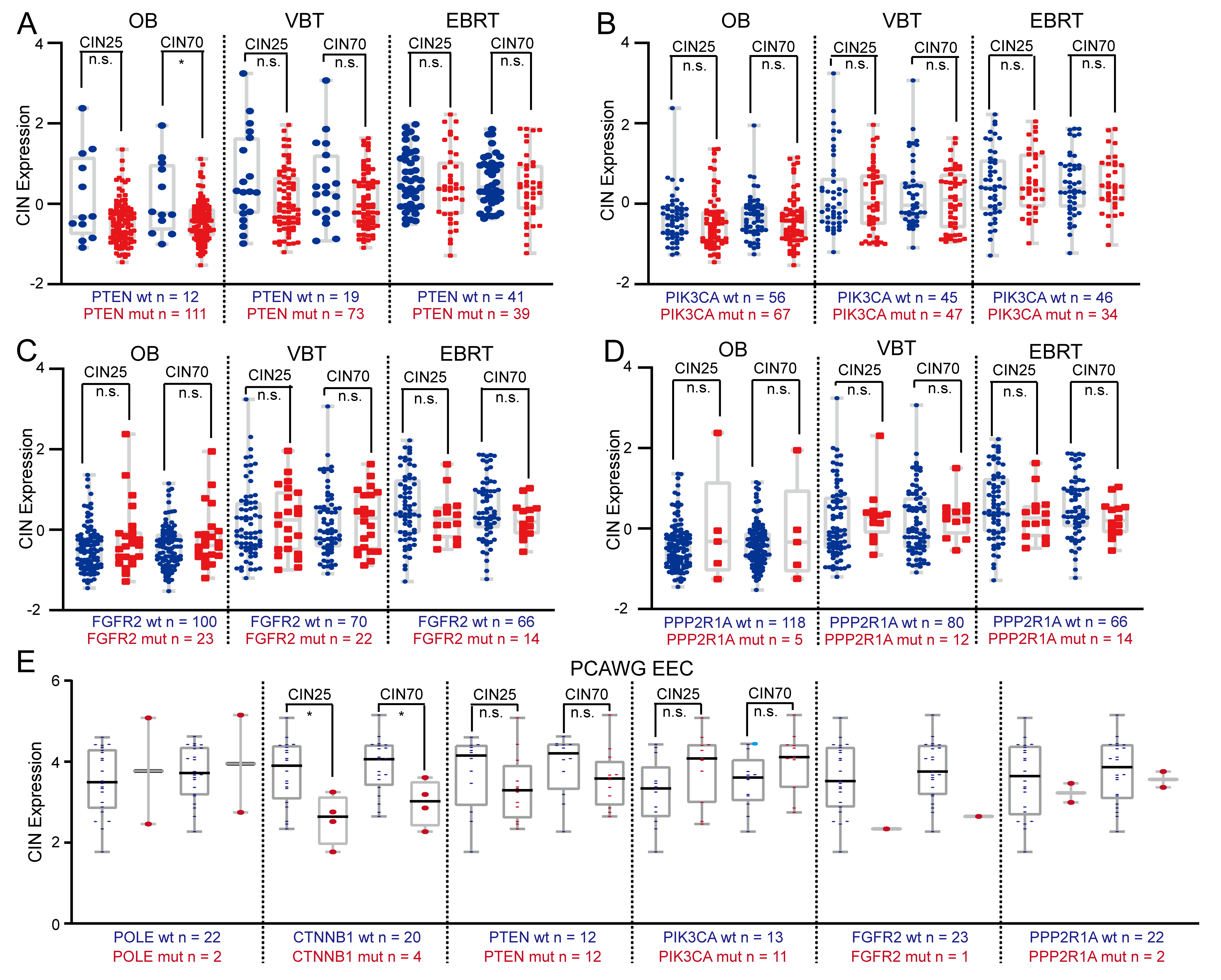

Supplement: Supplementary file 2 — Additional file 2: Figure S2. Relationships between CIN and molecular prognostic factors. a Boxplot of CIN25 and CIN70 expression in PTEN wild-type (blue) versus mutant patients (red) from the OB, VBT and EBRT groups. b Boxplot of CIN25 and CIN70 expression in PIK3CA wild-type (blue) versus mutant patients (red) from the OB, VBT and EBRT groups. c Boxplot of CIN25 and CIN70 expression in FGFR2 wild-type (blue) versus mutant patients (red) from the OB, VBT and EBRT groups. d Boxplot of CIN25 and CIN70 expression in PPP2R1A wild-type (blue) versus mutant patients (red) from the OB, VBT and EBRT groups. e Boxplot of CIN25 and CIN70 expression in wild-type POLE, CTNNB1, PTEN, PIK3CA, FGFR2 and PPP2R1A (blue) versus mutant patients (red) from the PCAWG EEC cohort. P values in (a)–(e) are Mann–Whitney test calculations (*p < 0.05, **p < 0.01, ***p < 0.001, n.s. not significantly different). [file 13578_2020_486_MOESM2_ESM.tif]

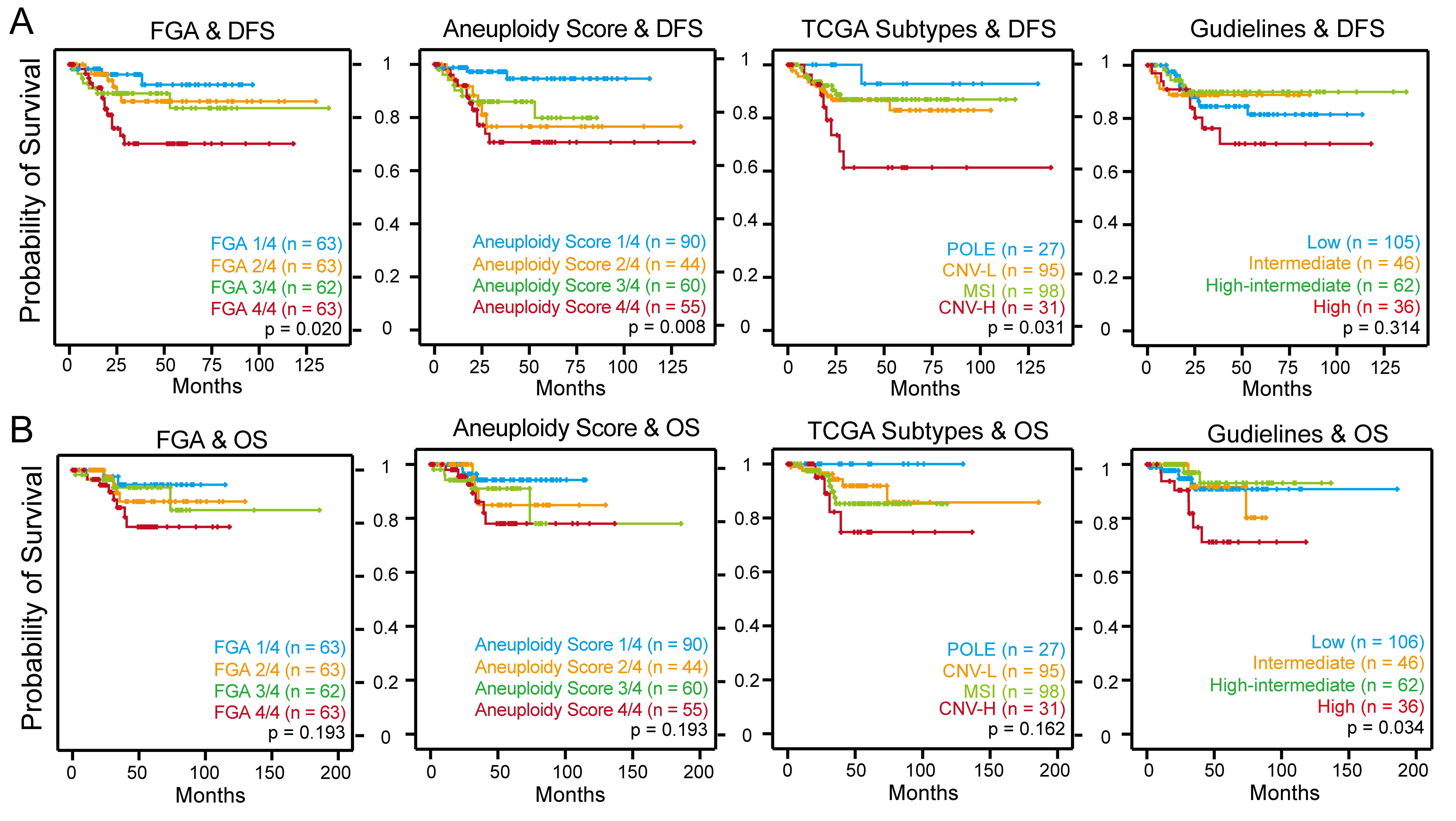

Supplement: Supplementary file 3 — Additional file 3: Figure S3. Kaplan–Meier plot for DFS and OS in Stage I EEC from TCGA based on Guidelines, FGA, Aneuploidy Score and TCGA subtypes. a 5-year PFS. b 10-year OS. Patients are grouped by quartiles of FGA and Aneuploidy Score. P values for (a) and (b) are calculations of the log-rank test. [file 13578_2020_486_MOESM3_ESM.tif]
